# An examination of the psychometric structure of the Multidimensional Pain Inventory in temporomandibular disorder patients: a confirmatory factor analysis

**DOI:** 10.1186/1746-160X-2-48

**Published:** 2006-12-14

**Authors:** Yolanda Andreu, Maria J Galdon, Estrella Durá, Maite Ferrando, Juan Pascual, Dennis C Turk, Yolanda Jiménez, Rafael Poveda

**Affiliations:** 1 Department of Personality, Assessment, and Psychological Treatment, University of Valencia, Spain; 2 Department of Methodology, Psychobiology and Social Psychology, University of Valencia, Spain; 3 Department of Anaesthesiology, University of Washington, US; 4 Service of Stomatology, University of Valencia General Hospital, Spain

## Abstract

**Background:**

This paper seeks to analyse the psychometric and structural properties of the Multidimensional Pain Inventory (MPI) in a sample of temporomandibular disorder patients.

**Methods:**

The internal consistency of the scales was obtained. Confirmatory Factor Analysis was carried out to test the MPI structure section by section in a sample of 114 temporomandibular disorder patients.

**Results:**

Nearly all scales obtained good reliability indexes. The original structure could not be totally confirmed. However, with a few adjustments we obtained a satisfactory structural model of the MPI which was slightly different from the original: certain items and the *Self control *scale were eliminated; in two cases, two original scales were grouped in one factor, *Solicitous and Distracting responses *on the one hand, and *Social activities *and *Away from home activities*, on the other.

**Conclusion:**

The MPI has been demonstrated to be a reliable tool for the assessment of pain in temporomandibular disorder patients. Some divergences to be taken into account have been clarified.

## Background

There has been a growing realisation that chronic pain is a complex phenomenon that consists of and is influenced by a wide range of psychosocial, behavioural and physical factors [1,2]. The complexity of chronic pain has led a number of authors to suggest that adequate treatment for chronic pain sufferers will depend on a better understanding of the pain sufferer and a comprehensive assessment of all relevant factors.

Temporomandibular disorders (TMDs) consist of a group of musculoskeletal problems affecting the temporomandibular joint and associated structures. These disorders represent a significant problem within the field of oral medicine, and are prevalent enough to constitute a public health concern. However, while Carlsson [3] has reported that as much as 93% of the population may show a sign and/or symptom of TMD during their lifetime, only 5–13% exhibit clinically significant symptoms such as pain or severe dysfunction. The aetiology of the disorder is highly controversial; rigorous studies need to be carried out using reliable and valid instruments of pain assessment to have a better understanding of the concrete mechanisms found at the base of TMD.

A large number of psychometric measures have been developed to assess chronic pain sufferers, and the West Haven-Yale Multidimensional Pain Inventory (MPI) [4] is one of the most frequently used instruments in this assessment [5]. The MPI was based on the cognitive-behavioural perspective on pain emphasising the important role of cognitive, emotional, and behavioural contributions to the pain experience and related disability. The initial study reporting on the development of the MPI included two samples of consecutive chronic pain patients recruited from pain patients evaluated at the West Haven Veterans Administration Medical Center in the United States [4]. The types of pain syndromes were disparate. The most frequent was back pain (36.4%) and over 80% of the original sample were male. Exploratory and confirmatory factor analyses were used in determining the specific scales for the sections of the MPI. It is composed of 52 items distributed in three sections. Section 1, the *Impact of pain in patients' life*, Section 2,*The responses of others to the patients' communications of pain*, and Section 3, *The extent to which patients participate in common daily activities*. The first section includes five empirically derived scales assessing: pain severity [*Pain severity*, 3 items] the amount of interference that patients believed the pain had on their lives [*Interference*, 9 items]; patients' perceptions of their control over their lives [*Self-control*, 2 items]; levels of affective distress [*Affective distress*, 3 items]; and patients' perceptions of the amount of support they received from signficant others [*Social support*, 3 items]. The second section contains three empirically derived scales that include patients' perceptions regarding how their significant others responded to them when they experienced pain: *Punishing responses *[4 items], *Solicitious responses *[4 items], and *Distracting responses *[6 items]. The third section includes four empirically derived scales: namely, performance of *Household chores *[5 items], *Outdoor work *[5 items], *Activities away from home *[4 items], and *Social activities *[4 items].

The MPI has been used in a large number of studies with diverse pain syndromes including the following: headache [6], fibromyalgia syndrome [7], pain associated with cancer [8], systemic lupus erythematosus [9], chronic pelvic pain [10], phantom limb pain [11], and whiplash disorders [12], among others. In addition to being used as an outcome measure in clinical studies, the MPI has been shown to be predictive of long-term disability [12,13] and has been used as the basis for identifying subgroups of chronic pain patients and subsequently matching treatment to patient group characteristics [14]. As far as TMD patients are concerned, several investigators have used the MPI for the assessment of TMD samples [15-17]. Also, research by Dahlstrom, Widmark and Carlsson [18] provides evidence of the utility of the MPI for patients suffering from TMDs in predicting treatment response. Even though the MPI has been used with TMD patients, no studies have examined the reliability and factor structure of the instrument in this specific population.

With regard to the psychometric properties of the original instrument, the MPI has shown a high level of internal consistency (Cronbach α above .60 on almost every scale) and an acceptable reliability test-retest (between .70 and .94) [4]. Previous studies on the structural validity of the instrument indicate that the original structure is generally replicated in the majority of cases [19,20]; however, some aspects differ from the original structure. Firstly, the factor loading of some items does not coincide exactly with the original. Secondly, the scales *Distracting responses *and *Solicitous responses *(section II) [19], and the *Activities away from home *and *Social activities *scale (section III) [19,20] were lacking independence from each other. Thus, these results suggest combining those scales in the comprehensive assessment of the patient with chronic back pain.

The MPI has also been translated and adapted to various languages including German [21], Dutch [22], Swedish [23], and Italian [24]. Confirmatory factor analyses have established the correspondence between the scales in the original American version and the adaptations mentioned above. Again, it has been pointed out that the factor loading of some items does not coincide exactly [20]. In these adaptations, the greatest amount of deviation from the original structure is in the third section. In the German [21] and Dutch [22] adaptations, the factor analysis in section three showed that the four original scales were reduced to three with *Activities away from home *and *Social activities *combined into a single factor.

There is also a Spanish adaptation [25] in a sample of 100 patients suffering from benign heterogeneous chronic pain: women comprised 82% of the study; the mean age of participants was 54.88; the average time period of pain suffering was 71.27 months; and the majority were suffering from back pain. However, this version shows some important limitations. Firstly, on the basis of exploratory data analysis, a 12 scale structure was obtained in which the internal consistency of three of the scales is clearly unsatisfactory (alfa de Crombach .10, .58 y .59, respectively). Therefore, the internal consistency of three of the twelve scales does not guarantee a good measure of the content to be evaluated. Secondly, the study does not define the translation process which was carried out. The absence of back translation in the adaptation procedure assumes an important deficiency in order to guarantee the equivalency between the Spanish and the original versions.

The first aim of this paper is to translate and adapt the MPI to Spanish achieving the maximum degree of equivalency between the versions. This adaptation requires a study of the structural properties of the instrument. Thus, a second objective involves executing confirmatory factor analysis of the MPI to test if the original structure proposed by the authors reproduces the same in our temporomandibular sample patients. We expect that the original structure of the authors is confirmed on the basis of two fundamentals: a) to carefully obtain the highest equivalence possible between the Spanish version and the original, b) the existence of adaptations to other languages in the European context basically confirming the original structure of the instrument in other heterogeneous samples of patients with chronic pain. Finally, a third objective corresponds to the evaluation of the internal consistency of the MPI scales in this sample.

## Methods

### Sample and Procedure

The initial sample consisted of 125 patients suffering from TMDs who were referred to the Stomatology Service at the General Hospital of Valencia. The age range was established between 15 and 70 years old. A stomatologist specialised in these disorders conducted a clinical examination on each of the patients following the Research Diagnostic Criteria for Temporomandibular Disorders (RDC/TMD) [26]; those that had previously received occlusal, physical, or pharmacological treatment were discarded. This led to the rejection of 11 cases from the initial sample (N = 125). The final sample consisted of 114 Caucasian patients. The mean age of the participants was 35 (SD = 14), and 89% (N = 101) were women. This distribution was similar to previous studies [3].

Once the patients had been selected, they were invited to participate in the present study and signed an informed consent form approved by the the Institutional Review Board. A psychologist conducted an interview and administered the Spanish version of the MPI.

### Development of the Spanish MPI version

This version was developed in three steps. Firstly, the MPI was independently translated by three psychologists using criteria to achieve a model as exact as possible to the English version regarding content and form. Likewise, these psychologists were urged to detect items whose content did not respond to the equivalent cultural criteria following the steps proposed by Van de Vijver and Hambleton [27]. Secondly, the previous version translated by Ferrer et al [25] and the three translations were analysed and subjected to dispute by two judges. As a result, a final version of the instrument was then translated back into English by a native translator. Finally, an objective expert in the field of psychology compared both versions and determined that no significant differences existed between them. The definitive Spanish version was then accepted.

The analysis of the items from the cultural point of view assumed that one of the items of section 3 was jointly considered atypical in the Spanish context and therefore eliminated in the definitive version. This decision had also been taken by Ferrer et al [25]. It deals with item 2, "*mow the lawn*" as one of the activities that the patient could do. Grass is not common in the majority of Spanish housing, so that item was far from coherent within our context. The same consideration concerning the type of typical housing in Spain led to modifying the literal translation of item 6 "*work in the garden*" for "*work in the garden or with plants*", since this activity would be the equivalent in our context.

### Statistical Analysis

In order to test if the original instrumental structure was reproduced in the sample of Spanish tempromandibular patients, a confirmatory factor analysis of each section of the MPI using EQS [28] was conducted. Structural equation models are made up of simultaneous equations containing observed and latent variables, and these models therefore constitute a system of prediction that includes multiple regression and factor analysis. In the terminology used in structural equation analysis, a latent variable is a factor that is hypothesised from the observed variables and can be affected by other variables or other factors.

Due to the small sample size, the primary estimation procedure of parameters was the Satorra-Bentler, considered the most robust estimator [29]. Statistical accuracy of the adjustments are based on the values of Satorra-Bentler χ^2^, the RMSEA, the Bentler-Bonnet normative and non-normative indexes (NFI, NNFI), and the index of comparative adjustment (CFI). Satorra Bentler Chi-Square (χ^2^) expresses the degree of fit with which the model proposes to reproduce the data observed. The higher the value is, the higher the discrepancy between the data observed and those expected by the model, and the significance of this index has to be above .05. Nevertheless, it is an index which is highly dependent on the number of subjects. RMSEA (Root Mean Square Error of Approximation) is the discrepancy between the population covariance matrix and the model. By convention, there is a good model fit if RMSEA is less than or equal to .05. More recently, it has been suggested that RMSEA ≤ .06 should be the cut-off for a good model fit [29]. NNFI (non-normed fit index) compares the proposed model with a null model in which the variables are independent, adjusting this value according to the degrees of freedom. It is one of the fit indexes which is less affected by sample size. NNFI close to 1 indicates a good fit, but it is not guaranteed to vary from 0 to 1. By convention, NNFI values below .90 indicate a need to respecify the model. Some authors [29] have used the more liberal cut-off of .80. CFI (comparative fit index) compares the existing model fit with a null model, which assumes that the latent variables in the model are uncorrelated (the "independence model"). CFI is penalised by sample size. CFI varies from 0 to 1. CFI close to 1 indicates a very good fit. CFI is also used in testing modifier variables (those which create a heteroscedastic relation between an independent and a dependent variable, in such a way that the relationship varies by class of the modifier). By convention, CFI should be equal to or greater than .90 to accept the model, indicating that 90% of the co-variation in the data can be reproduced by the given model.

In the event of an unsatisfactory fit with the model, the following parameters were examined: modification indexes for factor loadings, standard errors, standard residuals, the statistical significance of each parameter and square multiple correlation [29].

Finally, alpha de Cronbach was calculated to establish the internal consistency of the scales, and Pearson's correlations between the scales were obtained.

## Results

The solutions of the confirmatory factor analysis in each section are shown in Table 1. The indexes fit for the hypothesised model were not satisfactory in any of the sections.

**Table 1:** CFA Indexes of the original structure proposed by the authors.

	Section I	Section II	Section III
χ^2 ^Satorra-Bentler Degrees of Freedom	427.49 170 p = .00	107.65 62 p = .00	253.57 119 p = 00
RMSEA 90% Interval of Confidence	.131 (.115–.146)	.093 (.062–.120)	.113 (.090–.131)
Bentler-Bonnet normed fit index	.573	.668	.651
Bentler-Bonner non-normed fit index	.645	.767	.740
Comparative fit index	.663	.815	.772

Thus, a new analysis was performed section by section, making some modifications in the original structure as a result of the examination of parameters [29]. The resulting structures in the original can be seen in Table 2. The modifying criteria of the original model structure and the adjustments achieved for the newly tested structure are explained below, section by section.

**Table 2:** Comparison of the structures regarding the three sections.

**Section I Kerns et at, 1985**		**Section I Andreu, et al**	
Interference	2, 3, 4, 8, 9, 13, 14, 17, 19	Repercussion of pain	3, 4, 8, 9, 12, 13, 14, 16, 17
Pain severity	1,7, 12	Pain severity	1, 7
Social support	5,10,15	Social support	5, 10, 15
Affective distress	6, 18, 20	Affective distress	18, 20
Self-control	11, 16	Interference with daily activities	2

**Section II Kerns et at, 1985**		**Section II Andreu, et al**	

Solicitous responses	2, 5, 8, 11, 13, 14,	Support responses	2, 3, 5, 6, 8, 9, 11, 12, 13, 14
Distracting responses	3, 6, 9, 12		
Punishing responses	1, 4, 7, 10	Punishing responses	1, 4, 7, 10

**Section III Kerns et at, 1985**		**Section III Andreu, et al**	

Household chores	1,5,9,13,17	Household chores	1, 5, 6, 9, 13, 17,
Outdoor work	2, 6, 10, 14, 18	Taking care of the car	10, 14
Activities away from home	3, 7, 11, 15	Social and leisure activities	3, 4, 7, 8, 11, 12, 15, 16
Social activities	4, 8, 12, 16		

### Section I

Several items were eliminated due to the fact that value t associated to the coeficient of the factor over the item was not significant: item 6 (*Overall mood during the past week*), 11 (*Amount of control over life during the past week*), and 19 *(Affects friendships with other than family members*). The subsequent CFA produced the following adjustment indexes shown in Table 3.

**Table 3:** CFA Satisfactory Indexes in three sections.

	Section I	Section II	Section III
χ^2 ^Satorra-Bentler Degrees of Freedom	129.53 111 p = .11	90.35 71 p = .06	131.59 113 p = .11
RMSEA 90% Interval of Confidence	.04 (.00–.07)	.06 (.00–.08)	.04 (.00–.07)
Bentler-Bonnet normed fit index	.83	.76	.80
Bentler-Bonner non-normed fit index	.96	.91	.96
Comparative fit index	.97	.93	.97

As can be observed in Table 3, the significance of Satorra-Bentler χ^2 ^test is over .05, so the analysed model appears to be satisfactory. It is important to emphasise that the Bentler-Bonnet non-normalised index, and the comparative index are both over .95 confirming the structure of section I of the MPI – once the three items were eliminated.

Once the items are eliminated, Factor I fits well with the original *Interference *scale (Table 2). However, this factor also included an item from the original *Pain severity *scale (item 12) and another item belonging to the *Self-control *scale (item 16). The loading that both elements have on the factor are among the lowest. The highest loadings of this factor were found for items 14, 4, 9 and 3. Their content refers mainly to the change perceived in the satisfaction obtained in family and social environments. This scale was named *Repercussion of pain*, instead of *Interference*, the original name, because the item that explicitly deals with the interference loaded on another factor.

**Table 4:** Item-factor loading matrices for section I.

Items	I	II	III	IV	V
3. Affects ability to work	.73***				
4. Affects the amount of satisfaction from social activities	.74***				
8. Affects ability to participate in social activities	.59***				
9. Affects the amount of satisfaction from family related activities	.73***				
12. Amount of suffering experienced because of pain	.37**				
13. Affects family and marital relationships	.65***				
14. Affects the amount of satisfaction from work	1.0***				
16. Ability to deal with problems during the past week	-.25*				
17. Affects ability to do household chores	.67 (e.f)				
5. Supportiveness of spouse in relation to pain problem		.70***			
10. Amount of spouse worry regarding pain problem		.62***			
15. Degree of spouse attentiveness to pain problem		1.0 (e.f)			
1. Level of pain at the present moment			.84 (e.f)		
7. Severity of pain during the past week			.71***		
18. Degree of irritability during the past week				.82 (e.f)	
20. Amount of tension or anxiety during the past week				.75 ***	
2. Interference with daily activities					1.0 (e.f)

NOTE: *p ≤ .05 ** p ≤ .01 *** p ≤ .001;

e.f = effect fixed.

Factor II coincides completely with the original *Social support *scale (items 5, 10 and 15). Factor III, corresponds to the scale *Pain severity*, but it is defined by two of the three items from the original model, as we have already mentioned, item 12 loaded on the first factor. Factor IV, *Affective Distress *was defined with two items instead of three, since one of the items eliminated from the analysis (item 6) also belonged to this factor. As can be seen, factor II, as factor III and factor IV retained the names and content of the original scales. Finally, factor V composed only by item 2 was defined by its meaning *Interference with daily activities* (Table 2). 

The significant item-factor loading are presented in Table 4.

### Section II

The initial CFA performed on section II showed that the significant fit of the original MPI model was not satisfactory (Table 1). However, the modification indexes suggest that a reduction of the original number of factors. Two factors instead of the three original factors should respond better to the implicit structure present in the data. Besides, as noted in the introduction, in some previous studies that replicate the original structure, the data indicated the same results [19]. Therefore, an analysis was performed on a second model based on a bifactorial structure (Table 2) in which the original scales *Distracting responses *and *Solicitous responses *are combined in one. In this case, the indexes of adjustment of the modified structure (Table 3) indicate an overall acceptability of the model (Table 5).

**Table 5:** Item-factor loading matrices for section II.

Items	I	II
2 (II). Asks me how he/she can help	.51***	
3 (II). Reads to me	.45***	
5 (II). Takes over my chores	.65***	
6 (II). Talks to me to take my mind off the pain	.64***	
8 (II). Gets me to rest	.80 (e.f)	
9 (II). Involves me in activities	.38**	
11 (II). Gives me pain medication	.54***	
12 (II). Encourages me to work on a hobby	.57***	
13 (II). Gets me something to eat	.59***	
14 (II). Turns on the T.V.	.62***	
1 (II). Ignores me		.46***
4 (II). Expresses irritation to/at me		.73***
7 (II). Expresses frustration to/at me		.73 (e.f)
10 (II). Expresses anger to/at me		.76***

NOTE: *p ≤ .05 ** p ≤ .01 *** p ≤ .001;

e.f = effect fixed.

The first factor encompasses each and every one of the items that formed the original *Solicitous responses *and *Distracting responses *scales (2, 3, 5, 6, 8, 9, 11, 12, 13 and 14). Thus, the resulting factor was labelled *Support responses*. Furthermore, factor II includes the 4 items on the original *Punishing responses *scale (1, 4, 7 and 10). The original name of the factor was retained. The significant item-factor loading are presented in (Table 5).

### Section III

Because of the unsatisfactory fit of the original model in section III, some modifications were made. Item 18 (*Work on house repairs*), belonging to *Outdoor work *was eliminated from the model because of the value t factor coeficient on an item was not significant. Likewise, the modification indexes suggest that the implicit structure of the data responds to three factors instead of four. This structure of Section II based on three factors has also been found in previous papers that confirm the structure of the instrument [19-22], in which the original *Social activities and Activities away from home *scales are combined together. This three-scale structure was tested in a new confirmatory factor analysis (Table 2).

The indexes of adjustment and the significance test of the second tested model are shown in Table 3. The significance Satorra-Bentler χ^2 ^test is above .05, and the value of the adjustment indicators were satisfactory, with some of them even exceeding the value of .95. The significant item-factor loading are presented in Table 6.

**Table 6:** Item-factor loading matrices for section III.

Items	I	II	III
1. Wash dishes	.80***		
5. Go grocery shopping	.72***		
6. Work in the garden	.35**		
9. Help with the house cleaning	.85***		
13. Prepare a meal	.83***		
17. Do laundry	.84 (e.f)		
3. Go out to eat		.23*	
4. Play cards or other games		.55***	
7. Go to the cinema		.62***	
8. Visit friends		.61***	
11. Take a ride in the car		.68***	
12. Visit relatives		.63***	
15. Take a trip		.46 ***	
16. Go to the park or beach		.65 (e.f)	
10. Work on the car			.94***
14. Wash the car			.84 (e.f)

NOTE: *p ≤ .05 ** p ≤ .01 *** p ≤ .001;

e.f = effect fixed.

All the items in the original *Household chores *scale (1, 5, 9, 13 and 17) appear in factor I, so the initial name was retained. In addition, this scale includes item 6 (*Work in the garden or plants*), which originally belonged to the *Outdoor work *scale. It is worth mentioning that when this item was adapted to Spanish it was translated including the care of plants, an activity that is usually done inside the home. All the items obtained from the original *Social activities and Activities away from home *scales are grouped together in Factor II, the *Social and leisure activities *scale (3, 4, 7, 8, 11, 12, 15 and 16). Finally, factor III includes only those items referred to in *Taking care of the car *(10 and 14). In the original structure, those items were organised within the *Outdoor work *scale.

### Reliability

The internal consistency (Cronbach α) for each and every one of the MPI scales are satisfactory, exceeding the α of .70. (see Table 7) indexes.

**Table 7:** MPI obtained scales Internal Consistence.

Section I		Sectión II	
1. Repercussion of pain	.85	1. Negative responses	.78
2. Social support	.82	2. Support responses	.85
3. Pain severity	.75	Section III	
4. Negative mood	.75	1. Household chores	.80
5. Interference in daily activities	-	2. Social and leisure activities	.73
		3. Taking care of the car	.88

Finally, Table 8 depicts the correlations among the newly obtained scales. These results show a higher independence among the scales.

**Table 8:** Correlation between MPI obtained scales (N = 114).

	1(I)	2(I)	3(I)	4(I)	5(I)	1(II)	2(II)	1(III)	2(III)
2(I)	.13								
3(I)	.43***	.19							
4(I)	.19	-.10	.15						
5(I)	.67***	.17	.32**	.02					
1(II)	.29**	.32**	.24*	.14	.17				
2(II)	.17	-.32**	.11	.12	.06	-.20			
1(III)	-.15	-.13	-.07	.09	-.11	-.08	.15		
2(III)	-.12	-.04	-.19	.06	.01	.27**	-.16	.03	
3(III)	.04	.11	.09	.06	.04	.19	.07	.03	.42***

NOTE: *p ≤ .05 ** p ≤ .01 *** p ≤ .001

## Discussion

Although the results of the CFAs conducted did not completely confirm the original structure of the MPI, the structure resulting from our data with TMD patients in Spain appears to be highly consistent with the original proposal by Kerns et al [4]. Minor modifications were made including the elimination of several items. This occurred despite the fact that a heterogeneous sample (back pain being the largest percent) was used in the original study [4].

One of the differences between our results and the structure obtained by the authors of the original MPI psychometric paper [4] is the elimination of the *Self-control *scale. Several studies have found low reliability in this scale [20,21,23].

In section II, the results support the merging of both scales of positive responses – *Solicitous *and *Distractive responses *– into a single factor. Although some studies have worked out exactly the same structure for this section [19], others find a better refinement of the original model [20-24]. Characteristics of the samples used in the different studies, as well as cultural variants, may have a bearing on the conflicting results. In general, TMD patients do not show incapacitating pain and they manage better than patients with fibromyalgia syndrome, back pain, or migraine, with more presence in other samples [30]. In fact, for patients with TMD, pain is just one more aspect along with another symptomatology such as the reduction of the opening of the mouth and annoying mandibular sounds. These patients may perceive any positive response from the people in their environment as equally useful. It is not relevant whether that help comes in the shape of actions aimed towards distraction – *Distracting responses *– or in others openly channelled to the handling of the symptoms – *Solicitous responses*.

Supporting the results obtained in other studies [20,21,23], our data reproduce the structure of section III divided in three factors rather than four as originally proposed. We have grouped the *Social activities *and *Away from home activities *scales into a single scale. Another relevant feature of our outcome is the modification of the *Outdoor work *scale, in which the items exclusively related to *Taking care of the car *remain. Both cultural context and a sample made up of mostly women may contribute to this redefinition of the scale. This interpretation is supported in the previous Spanish adaptation of the instrument in patients with benign chronic pain [25].

In short, the aspects that characterize the MPI structure in the Spanish sample of temporomandibular patients are the elimination and change of some items in section I, and the combination of two of the original scales in a single one in section II and section III. Clearly, these aspects assume that there are differences regarding stuctural changes to the original model proposed by the authors. However, this paper is only a first approximation to the process of adaptation of an instrument to another language, which uses a specific sample of chronic pain. Future studies with different samples will be necessary to deal with the structural validity of the instrument in the Spanish context. This will allow us to declare reliability and stability of the obtained results.

## Conclusion

In summary, this paper supports the use of the MPI [4] for the assessment of temporomandibular patients showing satisfactory psychometric properties. Although the structure of the instrument in this sample shows some specific features to be considered, a complete line of investigation is required to consolidate the instrument adaption and validity to the Spanish population.
